# Screening of antigliadin and antitissue transglutaminase antibodies in patients with chronic plaque psoriasis: a case-control study

**DOI:** 10.55730/1300-0144.5615

**Published:** 2023-02-01

**Authors:** Burhan ENGİN, Çinare HÜSEYNOVA, Tümay AK, Ali Yağız AYLA, Günay CAN, Serdal UĞURLU

**Affiliations:** 1 Department of Dermatology, Faculty of Medicine, İstanbul University-Cerrahpaşa, İstanbul, Turkey; 2 Department of Internal Medicine, Faculty of Medicine, İstanbul University-Cerrahpaşa, İstanbul, Turkey; 3 Division of Rheumatology, Department of Internal Medicine, Faculty of Medicine, İstanbul University-Cerrahpaşa, İstanbul, Turkey; 4 Department of Public Health, Faculty of Medicine, İstanbul University-Cerrahpaşa, İstanbul, Turkey

**Keywords:** Psoriasis, celiac disease, antibodies

## Abstract

**Background/aim:**

In this study, we aimed to investigate different types of celiac antibodies in psoriasis patients and to see if the presence of the antibodies was associated with other variables.

**Materials and methods:**

We included patients with plaque psoriasis who were followed up in our dermatology clinic between February 2019 and February 2021 and added a healthy control group for comparison. The antibodies studied were serum antitissue transglutaminase (tTG)-IgA, tTG-IgG, antigliadin antibody (AGA)-IgA, and AGA-IgG. The patients’ records were used to note age, sex, the pattern of psoriasis involvement, psoriasis area and severity index (PASI), presence of hypertension, presence of type 2 diabetes mellitus, use of methotrexate, and use of biologic agents.

**Results:**

Sixty-five psoriasis patients (31 F, 34 M, mean age: 38.9 ± 15.2) and 65 controls (42 F, 23 M, mean age: 40.7 ± 13.2) were included in the study. There was no significant difference in antibody levels between the groups: tTG-IgA (2.4 U/mL vs 3.2 U/mL, p = 0.11), tTG-IgG (2.2 U/mL vs 3.2 U/mL, p = 0.74), AGA-IgA (2.4 U/mL vs 3.5 U/mL, p = 0.068), and AGA-IgG (3.2 U/mL vs 4.2 U/mL, p = 0.15). One patient from the psoriasis group only had borderline positive antibody levels whereas the rest of the psoriasis and control group had negative levels. Hypertensive psoriasis patients had significantly higher AGA-IgA titers compared to normotensive psoriasis patients (4.2 U/mL vs 2.3 U/mL, p = 0.005).

**Conclusion:**

There was no increase in the AGA-IgA/IgG and tTG-IgA/IgG levels in psoriasis patients compared to the healthy population. However, hypertensive psoriasis patients had higher AGA-IgA levels compared to normotensive ones.

## 1. Introduction

Psoriasis is a chronic, autoimmune dermatosis that is seen in more than 125 million people around the world [1]. It is most often characterized by erythematous plaques covered by silvery scales accompanied by systemic symptoms. As with other autoimmune diseases, the disease develops in patients with susceptible genetic predispositions and is set off by environmental triggers, although the exact triggers are usually unknown. The psoriasis-susceptibility (PSORS1) locus located on chromosome 6p21 was found to be a crucial factor in psoriasis development and HLACw6 is one of the most strongly implicated alleles in psoriasis [2, 3]. The interplay between TNF-α, IL-17, and IL-23 is at the core of psoriasis pathogenesis and thus the target of treatments [4]. Psoriasis can be accompanied by other immune-mediated diseases including psoriatic arthritis, rheumatoid arthritis, alopecia areata, and celiac disease [5].

Celiac disease (CD), also called celiac sprue or gluten-sensitive enteropathy, is an autoimmune disorder triggered by gluten and histologically characterized by villous atrophy, crypt hyperplasia, and intraepithelial lymphocytosis. The prevalence is estimated to be 1% in most populations [6]. The symptoms of CD include diarrhea, steatorrhea, weight loss, and flatulence. HLA-DQ2 and/or HLA-DQ8 are associated with CD development and their absence can be used to exclude the disease. However, the first-line test for CD diagnosis is the measurement of antitissue transglutaminase (tTG)-IgA. tTG-IgG, antiendomysial antibody (EMA-IgA), antigliadin antibody (AGA)-IgA, AGA-IgG, and deamidated gliadin peptide antibody (DGP-IgA, DPG-IgG) can also be used for screening or diagnosis in selected patients.

The efforts to investigate the coexistence of psoriasis and CD have led to different results. A 2020 metaanalysis found an odds ratio of 2.16 for CD in psoriasis patients and 1.8 for psoriasis for CD patients, both statistically significant [7]. Furthermore, some studies found an increased prevalence of celiac antibodies in psoriasis patients [8–10]. On the other hand, there are also studies that found no association between psoriasis and celiac antibodies [11, 12]. Therefore, no consensus has been reached regarding the association between psoriasis and CD markers. In this study, we aimed to look for different types of celiac antibodies in psoriasis patients to compare them with healthy controls. In addition, we aimed to see if any disease characteristics of the patients, including nonautoimmune comorbidities, differed according to the levels of celiac antibodies.

## 2. Materials and methods

This study included the psoriasis patients who were followed up at the Dermatology Clinic of Cerrahpaşa Medical School, İstanbul University-Cerrahpaşa between February 2019 and February 2021. The psoriasis diagnoses were made clinically by the characteristic plaque lesions and then confirmed histologically by the biopsy of the lesions. The patients under the age of 18 or who had psoriatic arthritis were excluded from the study. None of the patients had a CD diagnosis or any CD-suggesting complaints (abdominal pain, bloating and gas, constipation, diarrhea, nausea and vomiting, weight loss, and joint pain). Age- and sex-matched healthy controls were added for comparison. Both the patients’ and controls’ blood sera were measured quantitatively for tTG-IgA, tTG-IgG, AGA-IgA, and AGA-IgG using the AESKULISA® enzyme-linked immunosorbent assay (ELISA) test kits (AESKU.Diagnostics, Germany). The reference range for the antibody levels were as follows: <12 U/mL: negative; 12–18 U/mL: borderline positive; >18 U/mL: positive. Age, sex, the pattern of psoriasis involvement, PASI, presence of hypertension, presence of type 2 diabetes mellitus (T2DM), use of methotrexate (MTX), and use of biologic agents were noted.

The psoriasis group and the control group were compared in terms of age, sex, and antibody levels. Additionally, within the psoriasis group, antibody types and levels were compared between sexes, different patterns of skin involvement, patients having hypertension and patients who do not have hypertension, patients having T2DM and patients who do not have T2DM, and patients having different treatment regimens. The study was planned according to the Declaration of Helsinki and all patients gave their consent for their data to be used for research purposes when admitted to the dermatology clinic. Approval for the study was received from the independent ethics committee of Cerrahpaşa Medical before the initiation of the study.

All statistical analyses were performed with the IBM SPSS Statistics for Windows, v. 21.0 (IBM Corp., Armonk, NY, USA). Required sample size was calculated according to AGA-IgA, AGA-IgG, tTG-IgA, and tTG-IgG levels for the Mann–Whitney U test with a confidence level of 95% (p < 0.05) to achieve 80% power. Sexes were indicated as numbers and percentages, ages were indicated as mean plus standard deviation, and antibody levels were indicated as medians and interquartile ranges (IQRs). Sex distribution was evaluated by Pearson’s chi-square test. Age between the groups was compared using Student’s *t*-test. Antibody levels between the groups were compared using the Mann–Whitney U test. The correlation between two parameters including ages, antibody levels, and PASI scores was analyzed using Spearman’s rank correlation coefficient. A two-tailed p < 0.05 was considered statistically significant.

## 3. Results

There were 65 patients in the psoriasis group and 65 patients in the control group. The psoriasis group consisted of 31 females and 34 males, while the control group consisted of 42 females and 23 males (p = 0.052). The mean age of the psoriasis group was 38.9 ± 15.2, whereas the mean age of the control group was 40.7 ± 13.2 (p = 0.48). 55 (85%) patients had trunk involvement, 61 (94%) patients had extremity involvement, and 21 (32%) patients had scalp involvement. None of the patients had nail or palmoplantar involvement. Twenty-two patients received MTX for treatment and 7 of these patients also received topical treatment in addition to MTX. Nineteen patients received biological treatments (either anti-TNFα or anti-IL17A agent). Twenty-one patients received topical treatment only, and 3 patients received acitretin only.

There was no significant difference in the levels of tTG-IgA (p = 0.11), tTG-IgG (p = 0.74), AGA-IgA (p = 0.68), AGA-IgG (p = 0.15) between psoriasis and the control groups (Table 1). Only one patient in the psoriasis group had a borderline positive result for the antibodies tested (tTG-IgA, tTG-IgG, AGA-IgA, and AGA-IgG). Within the psoriasis group, tTG-IgA levels were 2.2 U/mL and 2.4 U/mL for males and females, respectively (p = 0.91). Similarly, the tTG-IgG levels were 2.2 U/mL and 2.5 U/mL for males and females, respectively (p = 0.89). On the other hand, the AGA-IgA levels were 3.3 U/mL and 2.3 U/mL for males and females, respectively (p = 0.46) and the AGA-IgG levels were 3.2 U/mL and 3.5 U/mL for males and females, respectively (p = 0.87).

The relationships between the presence of trunk involvement in psoriasis and the levels of tTG-IgA (p = 0.95), tTG-IgG (p = 0.91), AGA-IgA (p = 0.35), or AGA-IgG (p = 0.18) were not significant. Likewise, the relationships between the presence of scalp involvement and the levels of tTG-IgA (p = 0.50), tTG-IgG (p = 0.89), AGA-IgA (p = 0.28), or AGA-IgG (p = 0.56) were not significant either (Table 2).

Psoriasis patients who had hypertension had significantly higher AGA-IgA levels compared to those who did not have hypertension (4.2 U/mL vs 2.3 U/mL, p = 0.005). However, there was no difference in the levels of tTG-IgA (p = 0.55), tTG-IgG (p = 0.58), or AGA-IgG (p = 0.62) between the hypertensive and normotensive psoriasis patients. In addition, the relationships between T2DM and the levels of tTG-IgA (p = 0.82), tTG-IgG (p = 0.79), AGA-IgA (p = 0.49), or AGA-IgG (p = 0.20) were not significant (Table 3).

The differences in the levels of tTG-IgA (p = 0.11), tTG-IgG (p = 0.17), AGA-IgA (p = 0.51) AGA-IgG (p = 0.23) between psoriasis patients who used MTX and the control group were not significant. Similarly, there were no differences in the levels of tTG-IgA (p = 0.54), tTG-IgG (p = 0.49), AGA-IgA (p = 0.65) AGA-IgG (p = 0.40) between the psoriasis patients who used biological agents and the control group (Table 4). Also, there were no significant differences in the levels of tTG-IgA (p = 0.96), tTG-IgG (p = 0.29), AGA-IgA (p = 0.81) AGA-IgG (p = 0.53) between the psoriasis patients using MTX and psoriasis patients who did not use MTX. Lastly, no significant difference in the levels of tTG-IgA (p = 0.39), tTG-IgG (p = 0.10), AGA-IgA (p = 0.73), and AGA-IgG was found between the patients who used biologic agents and those who did not (p = 0.82) (Table 5).

We found significant correlation only between different types of antibodies: AGA-IgA was correlated with tTG-IgA (r = 0.611, p < 0.001), tTG-IgG (r = 0.532, p < 0.001), and AGA-IgG (r = 0.418, p = 0.001). AGA-IgG was correlated with tTG-IgA (r = 0.349, p = 0.004) and tTG-IgG (r = 0.436, p < 0.001). tTG-IgA was correlated with tTG-IgG (r = 0.844, p < 0.001). None of the other correlations were significant: PASI scores were not correlated with age (r = 0.160, p = 0.207), tTG-IgA (r = −0.075, p = 0.557), tTG-IgG (r = −0.071, p = 0.579), AGA-IgA (r = −0.004, p = 0.976), or AGA-IgG (r = 0.034, p = 0.788). Age was not correlated with tTG-IgA (r = −0.231, p = 0.064), tTG-IgG (r = −0.193, p = 0.123), AGA-IgA (r = −0.078, p = 0.539), or AGA-IgG (r = −0.061, p = 0.630).

## 4. Discussion

In the present study, we did not detect a significant increase in celiac antibody levels tested in psoriasis patients compared to the control group, and the antibody titers were not significantly different between the sexes. In addition, the antibodies were tested negative in all psoriasis patients, except for one whose antibody levels were borderline positive. We observed that AGA-IgA titers were significantly higher (4.2 vs. 2.3, p = 0.005) in psoriasis patients with hypertension than in normotensive patients. However, this study did not reveal a significant difference in celiac antibodies between the patients receiving biologic agents and MTX treatment and the control group. Within the psoriasis group, celiac antibody levels did not differ significantly in the patients who received and did not receive MTX therapy and those who received and did not receive biologic treatment. Celiac antibodies were not significantly related to specific involvement patterns of psoriasis, namely trunk or scalp. Although none of the antibodies were correlated with the severity of psoriasis (PASI scores) or patients’ ages, their levels were significantly correlated with each other as expected.

It has long been controversial whether there is a relationship between CD and psoriasis since scarce available data were inconsistent. Indeed, psoriasis appeared to be associated with an increased risk of several comorbidities, including metabolic syndrome, inflammatory arthritis, atherosclerotic disease, and inflammatory bowel diseases [13, 14]. First, in 1971, Marks and Shuster suggested that there might be a relationship between CD and psoriasis, and they thought that skin and intestinal diseases might be linked in different ways: (1) malabsorption can cause a rash; (2) a rash can cause malabsorption; (3) skin abnormalities and malabsorption can have a common cause; and (4) skin disease and malabsorption can be related indirectly [15].

A 2017 metaanalysis demonstrated that the risk of CD significantly increased among psoriasis patients compared to participants without psoriasis with an OR of 3.09 (95% confidence interval (CI): 1.92–4.97) [16]. A recent 2-way metaanalysis found significant odds ratios (OR) of 2.16 (95% CI: 1.74–2.69; 9 studies) for CD in patients with psoriasis and 1.8 (95% confidence interval, 1.36–2.38; 8 studies) for psoriasis in patients with CD [7]. They also found a significantly increased risk of new-onset psoriasis in CD (hazard ratio: 1.75; 95% CI: 1.58–1.93). However, subgroup analyses according to the disease severity and geographic region could not be performed [7]. On the other hand, a study from Iran found that the prevalence of CD in psoriatic patients was not more than that in the general population of Iran [17]. Considering the results of these studies and our study, racial factors, geographical conditions, and dietary habits might also affect CD prevalence among psoriasis patients.

Several controlled studies have supported the association between celiac biomarkers and psoriasis [8–10, 18–21]. However, these studies did not examine the potential effects of comorbidities, involvement patterns, or immunosuppressive treatments on celiac antibodies [8–10, 18–21]. Unlike our study, the fact that psoriasis patients with symptoms suggestive of CD were also included in these studies may have affected the results. On the other hand, smaller studies that did not employ control groups failed to demonstrate any relation between psoriasis and CD biomarkers [11, 12, 17, 22, 23]. Moreover, most of these studies only performed CD screening using AGA-IgA/IgG [11, 12, 17, 22, 23]. Endoscopic evaluation was performed for the patients with positive antibodies in CD screening in the studies mentioned above. However, in our study, endoscopic evaluation was not performed on any patient since there were no patients with positive antibodies in the psoriasis group or control group.

A metaanalysis performed across nine studies showed a statistically significant relative risk of having positive AGA-IgA in patients with psoriasis compared to controls: OR= 2.36, 95% CI: 1.15–4.83 [24]. Also, two studies suggested that the levels of celiac antibodies correlate with psoriasis or psoriatic arthritis severity [25, 26]. If there is a correlation between psoriasis severity and celiac antibodies, the role of celiac antibodies in the pathogenesis of psoriasis may be confirmed. However, as in our study, another case-control study did not show any significant correlation between celiac antibody level and psoriasis severity [27]. Still, they found that celiac disease patients had the highest AGA-IgA levels, followed by psoriasis patients with moderate levels and healthy controls with only weak positive AGA-IgA levels [27]. Likewise, another study from India found no relationship between age, sex, the severity of psoriasis, and celiac antibody levels [28]. Moreover, there are studies suggesting that a gluten-free diet may be beneficial in celiac antibody-positive psoriasis patients, but additional, better-designed studies are needed to confirm this [29–34]. In our study, we did not recommend a gluten-free diet to any participant since nobody was celiac antibody-positive.

The pathogenesis behind the increased risk of CD among patients with psoriasis is not well known, but there are different hypotheses [16]. Some at-risk HLA haplotypes are believed to play a role in the pathogenesis of both diseases. GWAS of these two diseases identified genetic susceptibility loci at eight genes that regulate innate and adaptive immune responses: RUNX3, TNFAIP3, ELMO1, ZMIZ1, ETS1, SOCS1, SH2B3, and UBE2L3 [35–38]. Another possible explanation is that intestinal barrier dysfunction associated with undiagnosed or untreated CD increases the gut permeability, thereby increasing the passage of immune triggers resulting in an enhanced risk of autoimmune diseases, including psoriasis [39, 40]. On the other hand, increased IL-1 and IL-18 derived from proliferating keratinocytes induce Th1 cells, and mucosal inflammation in CD is also caused by the activation of Th1 in response to dietary gluten [39]. Therefore, it is plausible that these ILs might predispose patients to CD. Finally, CD-related malabsorption, especially in severe cases, may worsen psoriasis through the deficient status of vitamin D, which is known to have immunoregulatory properties on psoriatic skin lesions [41]. In our study, we consider that there are several possible pathogenic mechanisms related to the significantly higher AGA-IgA level in hypertensive psoriasis patients compared to normotensive ones. Adaptive and innate immunity have been shown to be associated with arterial hypertension by increasing vessel wall stiffness [42]. Thus, the possibility of hypertension as a comorbidity increases in individuals with psoriasis and CD. Previously unrecognized common genome loci or HLA haplotypes may predispose to all three diseases. Hypertensive patients are likely to have poor gluten-rich dietary habits, which poses a risk of developing psoriasis and CD through the pathogenetic mechanisms mentioned above. In addition, individuals with this eating habit are generally prone to obesity, a known risk factor for psoriasis [43].

The most important limitation of this study was that we were not able to test anti-EMA IgA or IgG antibodies since we did not have their test kits in our hospital. However, we believe this study is an important contribution to the field, since there is no consensus on the relationship between psoriasis and CD, and the available data are limited. Moreover, searching whether there is a relationship between celiac antibody levels and comorbidities accompanying psoriasis, the involvement patterns of psoriasis, and the treatments used for psoriasis are the strengths of the study, which distinguish it from previous studies in the literature.

## 5. Conclusion

In this study, AGA-IgA/IgG and tTG-IgA/IgG were not significantly higher in psoriasis patients compared to the control group. Only hypertensive psoriasis patients had substantially higher AGA-IgA levels than normotensive psoriasis patients. In addition, celiac antibody levels did not differ significantly according to the involvement patterns of psoriasis and treatment modalities. However, we need more studies to confirm these results and comment on whether psoriasis patients should routinely be screened for CD.

## Figures and Tables

**Table 1:** Comparison of the psoriasis and control groups.

	Control group n = 65	Psoriasis group n = 65	p
Female sex, n (%)	42 (64.6%)	31 (47.7%)	0.052
Age, (mean ± SD)	38.9 ± 15.2	40.71 ± 13.2	0.48
Antigliadin IgA, median (IQR:25–75)	3.5 (2.3–5.4)	2.4 (1.8–4.3)	0.068
Antigliadin IgG, median (IQR:25–75)	4.2 (2.7–5.4)	3.2 (2.1–5.4)	0.15
Anti-TTG IgA, median (IQR:25–75)	3.2 (1.3–5.4)	2.4 (1.1–4.5)	0.11
Anti-TTG IgG, median (IQR:25–75)	3.2 (1.1–5.1)	2.2 (1.1–4.5)	0.74

**IQR**; interquartile range, SD; standard deviation, **TTG**; tissue transglutaminase.

**Table 2:** Comparison of celiac antibodies according to the involvement pattern of psoriasis.

	Trunk involvement		*p*	Scalp involvement		p
	Present a n = 55	Absent b n = 10		Present a n = 21	Absent c n = 44	
Antigliadin IgA, Median (IQR:25–75)	2.4 (2.0–4.3)	2.1 (1.0–4.3)	0.35	2.3 (1.7–4.4)	3.1 (1.6–4.2)	0.28
Antigliadin IgG, Median (IQR:25–75)	3.2 (2.2–5.5)	2.3 (1.2–4.6)	0.18	3.5 (2.2–5.1)	3.2 (2.1–5.3)	0.56
Anti-TTG IgA, Median (IQR:25–75)	2.2 (1.1–4.5)	2.4 (1.2–4.1)	0.95	3.2 (1.2–5.4)	1.9 (1.0–4.2)	0.50
Anti-TTG IgG, Median (IQR:25–75)	2.2 (1.0–4.8)	2.2 (1.8–4.1)	0.91	2.5 (1.6–4.7)	2.0 (1.0–4.5)	0.89

**IQR**; interquartile range, **TTG**; tissue transglutaminase.

a In this group, 13 patients had both scalp and trunk involvement.

b In this group, four patients only had extremity involvement, one patient only had scalp involvement, and five patients had both extremity and scalp involvement.

c In this group, 37 patients had trunk and extremity involvement, four patients had only extremity involvement, and three patients had only trunk involvement.

**Table 3:** Comparison of celiac antibodies according to comorbidities within the psoriasis group.

	Hypertension		*p*	Type 2 diabetes		p
	Present a n = 14	Absent b n = 39		Present c n = 15	Absent b n = 39	
Antigliadin IgA, median (IQR:25–75)	4.2 (2.3–8.8)	2.3 (1.5–4.1)	0.005*	3.1 (2.0–4.3)	2.1 (1.1–3.9)	0.49
Antigliadin IgG, median (IQR:25–75)	3.6 (3.0–5.9)	3.2 (2.1–5.2)	0.62	3.2 (2.2–5.3)	2.8 (2.1–6.6)	0.20
Anti-TTG IgA, median (IQR:25–75)	1.9 (1.0–4.8)	2.4 (1.2–4.3)	0.55	2.4 (1.2–4.5)	2.1 (0.4–3.3)	0.82
Anti-TTG IgG, median (IQR:25–75)	2.5 (0.8–4.4)	2.2 (1.1–4.5)	0.58	2.5 (1.0–4.5)	2.1 (1.2–5.1)	0.79

**IQR**; interquartile range, **TTG**; tissue transglutaminase.

a In this group, five patients with hypertension had type 2 diabetes mellitus and two patients had chronic obstructive pulmonary disease.

b This group includes patients with psoriasis and no other comorbidities.

c Five patients in this group had hypertension accompanying type 2 diabetes.

**Table 4:** Comparison of celiac antibodies between the psoriasis and control groups according to MTX and biological agent use.

	Methotrexate use a n = 22	Control group n = 65		Biologic agent use^b^ n = 19	Control group n = 65	p
Antigliadin IgA, median (IQR:25–75)	2.7 (2.1–4.5)	3.5 (2.3–5.4)	0.51	2.4 (2.0–4.6)	3.5 (2.3–5.4)	0.65
Antigliadin IgG, median (IQR:25–75)	3.0 (2.0–5.7)	4.2 (2.7–5.4)	0.23	3.2 (2.1–5.2)	4.2 (2.7–5.4)	0.40
Anti-TTG IgA, median (IQR:25–75)	2.3 (1.2–4.6)	3.2 (1.3–5.4)	0.11	3.1 (1.2–4.1)	3.2 (1.3–5.4)	0.54
Anti-TTG IgG, median (IQR:25–75)	2.1 (1.3–3.5)	3.2 (1.1–5.1)	0.17	4.1 (1.0–5.6)	3.2 (1.1–5.1)	0.49

**IQR**; interquartile range, **TTG**; tissue transglutaminase.

a In this group, 15 patients received only methotrexate, and seven patients received combined topical treatment with methotrexate.

b All patients in this group were treated with an anti-TNFα or anti-IL17A agent alone.

**Table 5:** Comparison of celiac antibodies according to treatment modalities of psoriasis.

	Methotrexate use		p	Biologic agent use		p
	Present a n = 22	Absent b n = 43		Present c n = 19	Absent d n = 46	
Antigliadin IgA, median (IQR:25–75)	2.7 (2.1–4.5)	2.4 (1.5–4.3)	0.81	2.4 (2.0–4.6)	2.4 (1.5–5.2)	0.73
Antigliadin IgG, median (IQR:25–75)	3.0 (2.0–5.7)	3.4 (2.1–5.2)	0.53	3.2 (2.1–5.2)	3.2 (2.2–5.5)	0.82
Anti-TTG IgA, median (IQR:25–75)	2.3 (1.2–4.6)	2.4 (1.0–4.2)	0.96	3.1 (1.2–4.1)	2.2 (1.0–4.5)	0.39
Anti-TTG IgG, median (IQR:25–75)	2.1 (1.3–3.5)	2.9 (1.0–5.1)	0.29	4.1 (1.0–5.6)	2.1 (1.2–4.2)	0.10

**IQR**; interquartile range, **TTG**; tissue transglutaminase.

a In this group, 15 patients received only methotrexate, and seven patients received combined topical treatment with methotrexate.

b In this group, 21 patients received only topical therapy, 19 patients received only biological therapy, and three patients received acitretin.

c All patients in this group were treated with an anti-TNFα or anti-IL17A agent alone.

d In this group, 15 patients received only methotrexate, and seven patients combined topical treatment with methotrexate, 21 patients only topical therapy, and three patients acitretin.

## Abbreviations

tTG: tissue transglutaminase
AGA: antigliadin antibody
EMA: antiendomysial antibody
PSORS: psoriasis susceptibility
HLA: human leukocyte antigen
DGP: deamidated gliadin peptide antibody
PASI: psoriasis area and severity index
T2DM: type 2 diabetes mellitus
CD: celiac disease
MTX: methotrexate
TNF: tumor necrosis factor
IL: interleukin
ELISA: enzyme-linked immunosorbent assay
IQR: interquartile ranges
CI: confidence interval
OR: odds ratio
GWAS: genome wide association study
